# A novel accurate LC-MS/MS method for quantitative determination of Z-lumirubin

**DOI:** 10.1038/s41598-020-61280-z

**Published:** 2020-03-10

**Authors:** Jana Jašprová, Aleš Dvořák, Marek Vecka, Martin Leníček, Ondřej Lacina, Petra Valášková, Miloš Zapadlo, Richard Plavka, Petr Klán, Libor Vítek

**Affiliations:** 1 Institute of Medical Biochemistry and Laboratory Diagnostics, Faculty General Hospital and 1st Faculty of Medicine, Charles University, Prague, Czech Republic; 2 HPST, s.r.o., Prague, Czech Republic; 3 Department of Pediatrics and Neonatology, Faculty General Hospital and 1st Faculty of Medicine, Charles University, Prague, Czech Republic; 4 Department of Chemistry and Recetox, Faculty of Science, Masaryk University, Brno, Czech Republic; 5 4th Department of Internal Medicine, Faculty General Hospital and 1st Faculty of Medicine, Charles University, Prague, Czech Republic

**Keywords:** Biochemistry, Health care

## Abstract

Although phototherapy (PT) is a standard treatment for neonatal jaundice, no validated clinical methods for determination of bilirubin phototherapy products are available. Thus, the aim of our study was to establish a such method for clinical use. To achieve this aim, a LC-MS/MS assay for simultaneous determination of *Z*-lumirubin (LR) and unconjugated bilirubin (UCB) was conducted. LR was purified after irradiation of UCB at 460 nm. The assay was tested on human sera from PT-treated neonates. Samples were separated on a HPLC system with a triple quadrupole mass spectrometer detector. The instrument response was linear up to 5.8 and 23.4 mg/dL for LR and UCB, respectively, with submicromolar limits of detection and validity parameters relevant for use in clinical medicine. Exposure of newborns to PT raised serum LR concentrations three-fold (*p *< 0.01), but the absolute concentrations were low (0.37 ± 0.16 mg/dL), despite a dramatic decrease of serum UCB concentrations (13.6 ± 2.2 *vs*. 10.3 ± 3.3 mg/dL, *p* < 0.01). A LC-MS/MS method for the simultaneous determination of LR and UCB in human serum was established and validated for clinical use. This method should help to monitor neonates on PT, as well as to improve our understanding of both the kinetics and biology of bilirubin phototherapy products.

## Introduction

**Figure 1:**
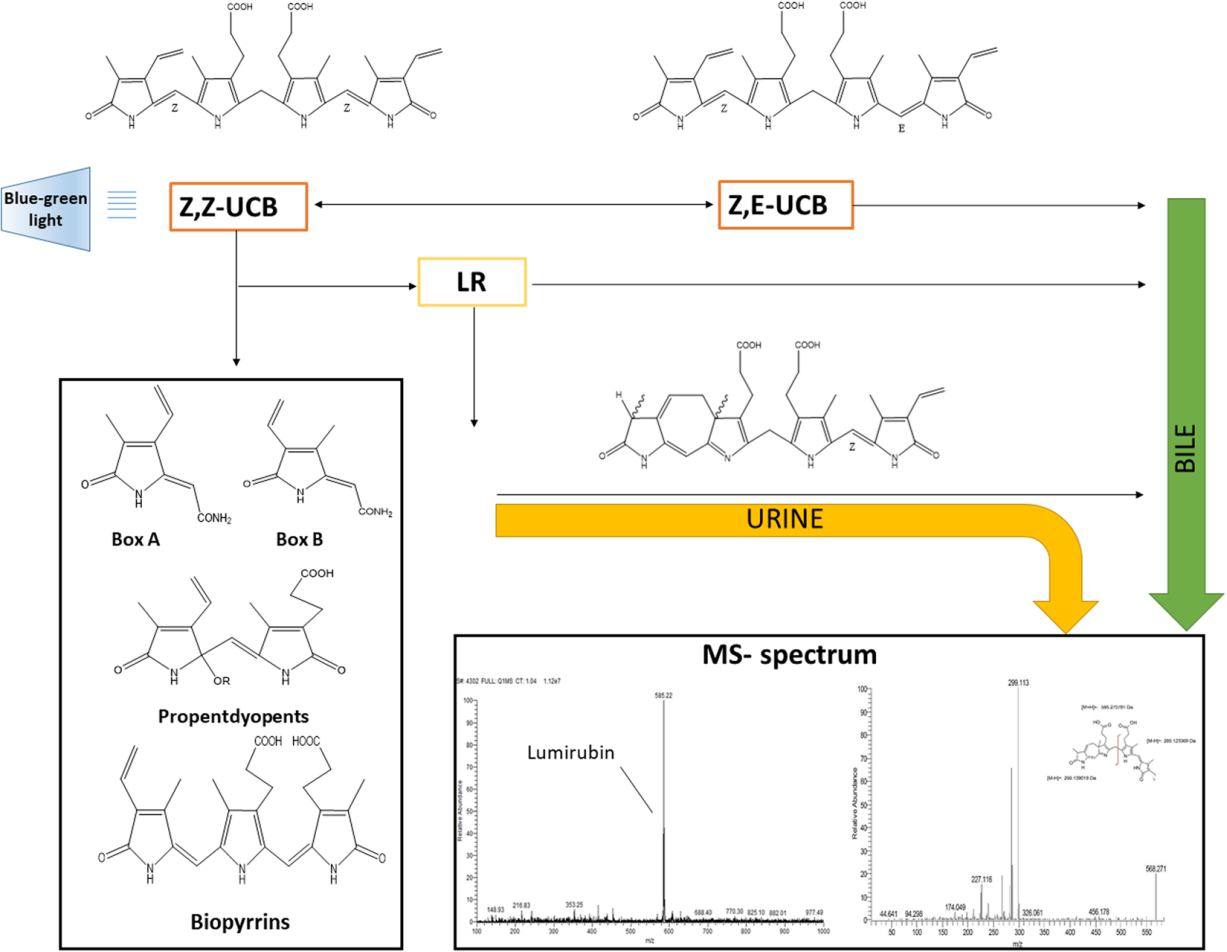
Bilirubin metabolism during phototherapy. The Figure depicts the fate of UCB during PT in the human body strengthening LR as the major PT product and indicating further major UCB oxidation products presumably produced during PT. MS spectrum shows maternal ion of LR (left side) and its MS/MS profile.

Neonatal jaundice is a very prevalent condition during the newborn period. In fact, virtually all newborn infants develop some degree of hyperbilirubinemia (>1 mg/dL, 17 μmol/L) during the first week of life. In up to one third of neonates, the total serum bilirubin concentrations exceed 13 mg/dL (220 μmol/L), depending on the geographical region as well as ethnicity^1–3^. Phototherapy (PT) with a blue-green light is the treatment of choice since its discovery in the 1950’s^4,5^. PT results in transformation of non-polar molecule of bilirubin into more polar derivatives - bilirubin photoisomers (and subsequently *Z*-lumirubin (LR) as the final product of the photo-rearrangement), and other bilirubin oxidation products (Fig. 1).

Although PT is used worldwide for decades in routine clinical practice, and in general this treatment modality is considered safe for neonatal infants^6^, certain health safety concerns have been raised recently, especially in extremely low-birth weight neonates. These include, in particular, increased mortality rates in those neonates treated with aggressive PT^7–9^. In addition, a significant increased risk of late-onset solid tumors in childhood infants treated with PT in the neonatal period has been reported recently^10^, a phenomenon likely related to DNA damage caused by blue-green light therapy PT^11^.

Based on this data, there is a need for a robust, accurate, and sensitive quantitative analytical method for the determination of bilirubin photoisomers; as well as photo-rearrangement and photooxidation products formed during PT. Quite surprisingly, these methods are still lacking, mainly because these bilirubin products are both chemically and photochemically unstable, and their determination requires a high level of expertise. In addition, standards of neither the bilirubin photoisomers nor the other photoproducts are commercially available which further complicates establishment of an appropriate clinical chemistry method. In fact, only scarce data on the preparation of these bilirubin derivatives were published in the scientific literature, with different purities and yields^12–15^.

Up to now, only two HPLC methods for LR determination have been reported so far. The first one was published by McDonagh and co-workers^16^ in 1982; but this method was not quantitative with only limited resolution of the separated bilirubin photoisomers. The latter was based on the correction of the HPLC chromatogram peak areas according to the different relative molar absorption coefficients of bilirubin photoisomers, but this method was not tested on clinical samples^17^. Importantly, these methods did not use the pure standards of bilirubin photoisomers and internal standard (ISTD) was lacking in these assays as well.

Therefore, the goal of our study was to establish and validate a robust analytical method for the LR quantification in samples of clinical neonatal serum.

## Materials and Methods

### Chemicals and reagents

Unconjugated bilirubin (UCB), human serum albumin (HSA), rabbit serum albumin (RSA), chloroform, dimethyl sulfoxide (DMSO), L-ascorbic acid (99%), and 2,6-di-*tert*-butyl-4-methylphenol (BHT) (≥99%) were all purchased from Sigma-Aldrich (MO, USA). Prior to use, UCB was purified according to McDonagh and Assisi^18^. The mesobilirubin (MBR) was from Frontier Scientific (UT, USA). The methanol for LCMS was from Biosolve Chimie SARL (France), and the ammonium fluoride LCMS additive (NH_4_F) was from Honeywell (International Inc., Morris Plains, NJ, USA). The ammonium acetate and natrium hydroxide were from Penta (Czech Republic), as were all the other common chemicals.

### LR preparation

Because of light sensitivity of both UCB and LR, all of the procedures were carried out under dim light in flasks wrapped in aluminum foil. The LR was prepared as described previously^15^, with slight modifications. Briefly, 2.8 mg of UCB was dissolved in 2 mL of 0.1 mol/L NaOH, immediately neutralized with 1 mL of 0.1 mol/L H_3_PO_4_, and mixed with 7 mL of 660 µmol/L RSA in PBS (RSA was used because it gives substantially higher LR yields compared to other albumins^19^). The final concentration of UCB was 480 µmol/L. The whole mixture was transferred to a Petri dish (10 cm diameter) and irradiated at 460 nm using a Lilly phototherapeutic device (TSE, Czech Republic) for 120 min at 70 μW/(cm^2^ nm), corresponding to a total irradiance of 2.2 mW/cm^2^. The irradiated bilirubin solution was deproteinated with 30 ml (1:3) of 0.1 mol/L ammonium acetate in methanol (pH change affects the structure of albumin and improves its precipitation) and then vortexed. Following the Folch extraction protocol^20^, chloroform (20 mL of chloroform per 20 mL of irradiated bilirubin solution in basic methanol, 1:1) was added and shaken intensively. Then, 10 mL of water was added to this mixture, vortexed, and centrifuged (10 min, 3,000 × g, 4 °C). The lower chloroform phase containing LR was transferred to the glass flask, and chloroform was distilled off at 40 °C on a vacuum rotary evaporator (RVO 200 A, INGOS, Czech Republic). The residue was dissolved in 300 µL of a chloroform/methanol solution (4:1, v/v), centrifuged at 5,000 x g for 5 min to eliminate residual impurities, and then separated by thin-layer chromatography on silica gel plates (PLC Silica gel 60, 0.5 mm 20 × 20 cm, Sigma-Aldrich) using a mobile phase of chloroform/methanol/water, 40:9:1, v/v/v. The yellow band corresponding to LR (verified by HPLC according to McDonagh^16^ and by LC-MS/MS) was scraped out of the plate, extracted by methanol, and dried under a stream of nitrogen at 60 °C.

The crude LR was dissolved in a HSA solution (400 μmol/L in PBS), and its concentration was spectrophotometrically determined at 453 nm (TECAN Infinite M200 spectrophotometer, Tecan Group Ltd., Switzerland) in multiple LR dilutions. The LR molar absorption coefficient was 33,000 mol^−1^ · dm^3^ · cm^−1^ as described earlier^15^. The purity of an isolated LR standard was 94% as evidenced by HPLC analysis (data not shown).

### Preparation of calibrators

The LR was diluted with HSA to the final concentration of 200 μmol/L and mixed with UCB dissolved in DMSO at a concentration of 800 μmol/L (1:1, v/v, stock solution). This stock solution was subsequently diluted by HSA to the final concentrations of 0.01, 0.1, 1, 10, 25, 50, and 100 μmol/L of LR; and to 0.04, 0.4, 4, 40, 100, 200 and 400 μmol/L of UCB. All calibration solutions were stored at −80 °C and used within a 3-month period. ISTD was prepared by dissolving commercial MBR in DMSO at the concentration of 0.5 mol/L. Stock solution was stored at −80 °C in 10 µL aliquots. Due to MBR instability, one aliquot was always used *per* only one experiment. Each aliquot was diluted with methanol (LC-MS quality) to a final concentration (5 µmol/L) just before use. Under these conditions (−80 °C, storage less than 6 months), MBR is sufficiently stable as tested in our previous study^21^.

Ten μL of HSA (blank) or LR/UCB and 10 μL of ISTD were mixed together and prepared for LC-MS/MS analyses. LR and UCB stabilities in the dark of the stored aliquots were tested during the entire process of method validation. We also specifically tested LR and bilirubin stability in human serum stored at −80 °C for three months. The adjustment for the endogenous level of bilirubin was performed by standard addition of bilirubin to the final concentration (40 μmol/L), possible presence of endogenous LR was neglected, since this could not affect the final concentration of LR.

### LC-MS/MS analysis

LC-MS/MS analysis was performed using a LC-MS platform^22^ with minor modifications. The HPLC system (Dionex Ultimate 3000, Dionex Softron GmbH, Germany) was equipped with a Poroshell 120 EC-C18 column (3.0 × 100 mm; 2.7 µm, Agilent, CA, USA). The analytes were detected in a triple quadrupole mass spectrometer (TSQ Quantum Access Max with HESI-II probe, Thermo Fisher Scientific, Inc., USA) operating in a positive SRM mode. The heated HESI-II probe for the MS detector was run under the following conditions: spray voltage +3,200 V, vaporizer temperature 350 °C, sheath gas 40 arbitrary units (au), auxiliary valve flow 15 au, ion sweep gas pressure 5.0 au, capillary temperature 320 °C. Skimmer offset voltage was not used. The tuning of MS/MS transitions was performed by the combined infusion of analytes (10 mg/L in the initial mobile phase, 20 µL/min) and the mobile phase (400 µL/min); the collision gas (Ar) pressure was set to 0.2 Pa. For targeted quantitative analysis of compounds (including ion confirmation using one quantifier and one qualifier transition), the monitored transitions (corresponding collision energy) were as follows: UCB [585.3 → 299.1 (20 V); 585.3 → 271.2 (18 V)], LR [585.3 → 299.1 (20 V); 585.3 → 285.1 (18 V)], and MBR [589.3 → 301.1 (20 V); 589.3 → 273.2 (44 V)]. Tube lens voltage was set at 83 V for UCB, 112 V for LR, and 85 V for MBR.

### Analysis of LR and UCB in the clinical serum samples

The clinical potential of the established method was assessed on clinical samples of newborn infants with neonatal jaundice treated with standard PT according to the current guidelines^23^ (PT apparatus FL 2010 (Alfamedic, Czech Republic)), the wavelength range of 425–475 nm with the peak at 446 nm, light intensity at 50 cm distance 58 μW/(cm^2^ nm). Blood samples were taken from 10 neonates on PT (before and a day following the initiation of PT).

The serum samples (10 μL) were mixed with 10 μL of ISTD. Deproteinization was performed after vortex-mixing of samples with 1 mL methanol containing 0.3% BHT, 0.1% ascorbic acid, and 0.5% ammonium acetate; with subsequent centrifugation for 40 min at 16,000 × g. One hundred μL of the final supernatant was carefully taken, and 3 μL were injected into a LC-MS/MS platform.

Chylous serum samples were obtained in the Central Biochemical Laboratory of the Faculty General Hospital, and anonymously used in our experiments. To obtain hyperbilirubinemic serum, a normobilirubinemic serum sample of a healthy volunteer was used, and spiked with 10 mM UCB dissolved in DMSO to make its final concentration of 18.4 mg/dL (315 μmol/L). To obtain a hemolytic serum, a volunteer whole blood sample was frozen at −80 °C and immediately thawed, then it was vigorously shaken and centrifuged at 3,000 × g for 10 min.

The collection and use of blood samples was approved by the Ethical Committee of the General University Hospital in Prague, Czech Republic and complied with the Helsinki Declaration of 1975 as revised in 2013. Informed consent was obtained from the parents of the examined neonates, as well as from the patients and volunteers for testing of possible interferences.

### Determination of UCB and LR kinetics in human serum exposed to continuous PT

Human serum from adult healthy volunteer was spiked with 10 mM UCB to the final concentration of 18.4 mg/dL (315 µmol/L). The whole mixture was transferred to a Petri dish and was then irradiated for 6 h, as described above for the experimental irradiation studies. Samples of the irradiated solutions were collected every 60 min. Ten µL of serum were pipetted into 1.5 ml Eppendorf plastic tubes which were immediately stored at −80 °C. ISTD was added before analysis, then the samples were processed, and the UCB and LR were determined as described above.

### Statistical analysis

Concentrations of UCB and LR in the neonatal sera were compared by the paired t-test. Differences were considered statistically significant when *p*-values were <0.05. Statistical analyses were performed using Prism 5.03 software (GraphPad, CA, USA).

## Results

### LC-MS/MS analysis

**Figure 2:**
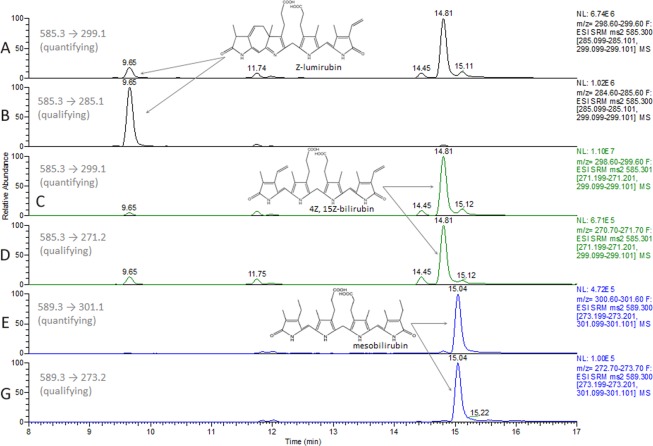
LC-MS/MS chromatograms of LR and UCB in serum. (**A**,**B**) LR (0.58 mg/dL; 10 µmol/L), retention time (RT) = 9.66 min; (**C**,**D**) UCB (2.33; 40 µmol/L), RT = 14.80 min; and (**E,F**) and MBR (ISTD), RT = 15.04 min are depicted by arrows. Smaller peaks close to RT of UCB (14.45 min and 15.11) represent UCB IIIα and XIIIα isomers. chromatograms are presented as intensities of selected quantifying and qualifying MRM transitions. ISTD, internal standard; LR, lumirubin; MRM, multiple reaction monitoring; UCB, unconjugated bilirubin; MBR, mesobilirubin; RT, retention time.

A binary mobile phase system consisting of 1 mmol/L of NH_4_F in water (A) and methanol (B) was used for sample separation. The flow rate was set at 0.4 mL/min and a column chamber was heated to 30 °C. The initial composition of mobile phase was 40% of B held for 3 min which was followed by a change to 100% B over 10 min, and kept for an additional 4 min. The proportion of phase B was switched to 60% in 0.1 min (17 → 17.1 min) and kept at the same value until the end of the gradient program at 20 min. Then, the content of the B phase turned back to initial conditions within 1 min, and the system was allowed to equilibrate for 4 min. To prevent the contamination of the MS, the flow from the HPLC was allowed to the detector between the 2^nd^ and 21^st^ min only. Typical chromatograms of LR and UCB in the serum are given in Fig. 2. (A-B for LR; C-D for UCB; and E-F for ISTD).

### Method validation

#### Robustness of the method

Serum samples, spiked with the standards of UCB and LR, were prepared once and frozen at −80 °C. Concentrations of LR and UCB were determined every month with practically no deterioration of the samples during the 3-month follow-up (Supplementary Fig. 1).

The stability of LR, UCB, and ISTD in the dark was tested after extraction by methanol, with or without added antioxidants, using a temperature-controlled autosampler (to simulate a delay in LC-MS measurements of the samples). The serum samples at three UCB and LR concentration levels (4, 40, and 400 µmol/L of UCB, paired with concentrations of LR 1, 10, and 100 µmol/L) were gradually prepared, extracted with methanol containing antioxidants, and were repeatedly injected during 6 h (Supplementary Fig. 2A,B). The samples (extracted by methanol) with 1 µM of LR and 4 µM of UCB were used to assess the effect of antioxidants on analyte stability. A dramatic increase of the LR and UCB concentrations was observed during analyses without antioxidants (5 and 2.7 times, respectively); this most likely due to the very fast degradation of ISTD, with a slower degradation rate of LR and UCB, which is consistent with our previous observations^21^. However, the addition of antioxidants (0.3% BHT, 0.1% ascorbic acid) improved the stability of ISTD as well as LR and UCB with no significant degradation observed during the 6 h testing (Supplementary Fig. 2A,B).

#### Linearity, limits of detection, and quantification of the method

**Figure 3:**
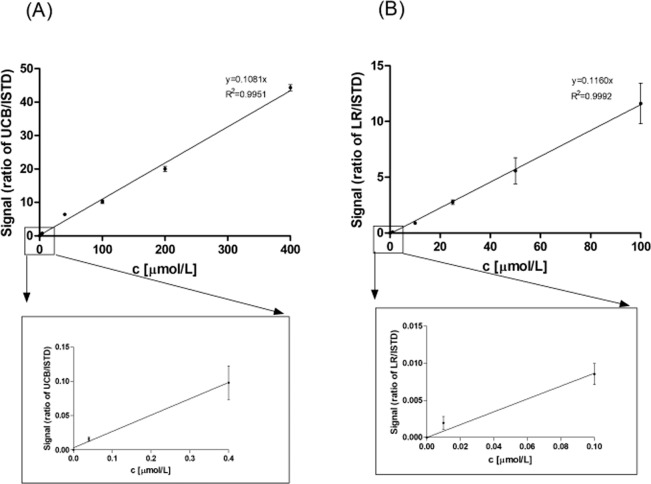
Calibration curves of UCB and LR. (**A**) Calibration curve of UCB. (**B**) Calibration curve of LR. Linearity was tested using 8 calibration points in triplicates. ISTD, internal standard; LR, lumirubin; UCB, unconjugated bilirubin.

The method was found to be linear within the ranges of 0.01 − 100 µmol/L and 0.04 − 400 µmol/L for LR and UCB, respectively (Fig. 3).

The limit of detection was estimated as the concentration that gives a signal 3 times above noise; whereas the limit of quantification was considered as a concentration that gives a signal 10 times above noise. As both limits were too low (limits of detection: 100 and 80 pmol/L for LR and UCB, respectively; limits of quantification: 330 and 264 pmol/L for LR and UCB, respectively) to be relevant for clinical practice, and fell below the tested linear range, the concentration of 1 μmol/L (a clinically relevant, and thus the lowest concentration used in the intra- and interassay imprecision analyses) was considered as a limit of quantification.

#### Intraassay/interassay imprecisions and average recoveries

The intraassay and interassay imprecisions, as well as average recoveries were within the acceptable range for both UCB and LR (Table 1). Specifically for UCB, intraassay and interassay imprecisions were within the range of 6.5–11% and 9.9–25%, respectively, while the same parameters for LR were within the range of 13–15% and 21–29%, respectively, for the concentrations used (UCB 4–400 μmol/L and LR 1–100 μmol/L) (Table 1).

**Table 1:** Intraassay and interassay imprecisions, and average recovery in clinically relevant UCB and LR concentrations.

UCB [µmol/L]	Intraassay Imprecision CV [%]	Interassay imprecision CV [%]	Average recovery ± SD [%]	Monitored transitions (m/z)	Expected ion ratio
4	11	25	118 ± 30	*585.3* → *299.1 (quantifying)*	1.3 (±15%)
40	18	20	108 ± 20		
400	6.5	9.9	101 ± 5	*585.3* → *271.2 (qualifying)*	
**LR [µmol/L]**					
1	13	21	93 ± 32	*585.3* → *299.1 (quantifying)*	16.4 (±15%)
10	14	29	78 ± 12		
100	15	27	101 ± 4	*585.3* → *285.1 (qualifying)*	

Intraassay imprecision was measured within one day; CV for 10 measurements of 3 specimens representing 3 spiked concentration levels (see the Stability assay in the Materials and Methods section) was calculated as SD/mean.

Interassay imprecision was measured over a 2 month period, with CV calculated for 10 measurements of 3 specimens.

The average recovery was calculated as [(measured concentration-initial concentration)/added concentration] for levels of the monitored analytes used in assay imprecision (n = 10).

CV, coefficient of variation; LR, lumirubin; SD, standard deviation; UCB, unconjugated bilirubin.

#### Interference testing

Neither high concentrations of bilirubin, triacylglyceroles, nor hemoglobin interfered significantly with the determination of UCB or LR, with recoveries between 92–108% (Table 2).

**Table 2:** Recoveries of known concentrations of UCB and LR in serum.

Interferences	UCB	LR
Hyperbilirubinemic serum^a^	NA	98.1 ± 3.9
Chylous serum^b^	91.8 ± 9.1	103.5 ± 9.4
Hemolytic serum^c^	107.8 ± 10.5	92.4 ± 11.2

^a^Bilirubin = 18.4 mg/dL (315 μmol/L); ^b^Triacylglyceroles > 151 mg/dL (1.7 mmol/L); ^c^Hemoglobin = 12 g/L

Recovery data given in % ± SD.

NA, not applicable; LR, lumirubin, UCB, unconjugated bilirubin.

Interferences were estimated as the recovery of a known amount of analyte added to the human serum samples (n = 6) containing various interferents.

### Determination of LR in clinical serum samples

**Figure 4:**
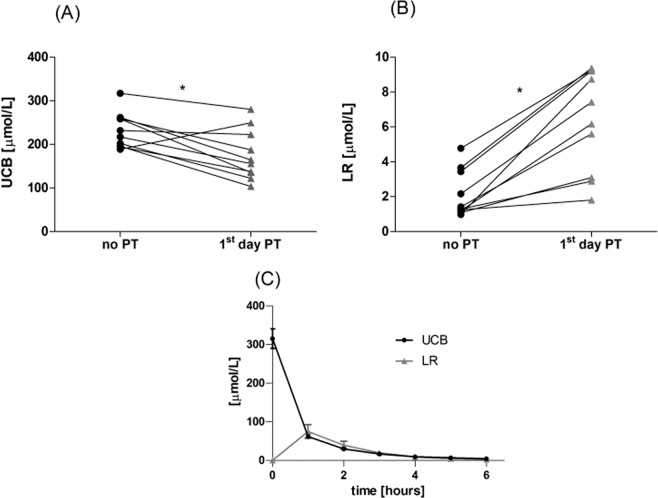
Serum concentrations of UCB (**A**) and LR (**B**) in neonates before and after PT, and (**C**) in human serum exposed to continuous PT. No PT means neonates with neonatal jaundice before PT. *p < 0.01. LR, lumirubin; UCB, unconjugated bilirubin.

The mean UCB concentrations of phototherapy-treated neonates decreased from 13.6 ± 2.2 mg/dL (233 ± 37 μmol/L) before the treatment to 10.3 ± 3.3 mg/dL (176 ± 56 μmol/L) the 1^st^ day after initiation of PT (*p* < 0.01, Fig. 4). This treatment resulted in a significant increase of the LR concentrations (0.12 ± 0.07 mg/dL (2.1 ± 1.2 μmol/L) to 0.37 ± 0.16 mg/dL (6.4 ± 2.7 μmol/L), *p* < 0.01), although it did not correspond to a marked decrease in the UCB concentrations.

### UCB and LR kinetics in human serum exposed to continuous PT

Because the LR concentrations detected in the serum samples of human neonates treated by PT were surprisingly low, we performed an *in vitro* study with human serum from a healthy volunteer that was spiked with UCB, reaching the final concentration of 18.4 ± 1.3 mg/dL (315 ± 22 μmol/L) (the mean of 4 technical replicates). The concentrations of LR and UCB in the samples of serum exposed to blue light for 6 h are demonstrated in Fig. 4C. The data clearly demonstrates a striking increase of the LR concentrations after 1 h of irradiation (4.39 ± 0.9 mg/dL, 75 ± 15 μmol/L) which rapidly decreased upon continuing irradiation, reaching single micromolar concentrations after 6 h. On the other hand, the UCB concentrations decreased dramatically and continuously during the whole irradiation period (Fig. 4C).

## Discussion

Although PT has become the gold standard therapy for severe neonatal jaundice^23^, surprisingly there is very limited knowledge about the biology of bilirubin photoproducts generated during PT. The major reason for this is the absence of a robust and reliable analytical method, which would be applicable for both basic and translational research as well as for use in clinical practice.

Difficulties in establishing such an analytical method are most importantly related to the low stability of bile pigments and their photodegradation products, resulting in both preanalytical as well as analytical problems. Simultaneously, the low chemical stability of primary and secondary bilirubin photoproducts is also a reason for the lack of commercially available analytical standards.

In our current study, we describe a much improved, analytical LC-MS/MS method for simultaneous determination of LR, the major bilirubin photoisomer; and UCB, with a sufficient sensitivity and accuracy to be used in clinical research. For this method, we used a standard of LR, whose isolation was originally described by McDonagh^15^ and modified in our previous *in vitro* studies^24^. Despite this achievement and all the effort made, it must be noted that isolated LR is still not sufficiently pure, and this is one of the reasons for suboptimal variability in the LR concentrations measured in clinical serum samples (the other reason is related to the chemical instabilities of the analytes). Thus, a LR standard, prepared by chemical synthesis (currently being researched in our laboratories) is definitely one of the major ways to solve this problem.

Nevertheless, accuracy of LR determination can be substantially improved by the addition of BHT and ascorbic acid in the proper ratio, as previously described^25,26^ to prevent oxidation of the pigments. The samples are stable at −80 °C for at least 3 months. However, they degrade fast during sample preparations and analyses, unless treated properly with antioxidants.

Previously, only one qualitative^16^ and one quantitative^17^ HPLC methods have been reported in the literature, but none of these was suitable for MS analysis because the solvents (in particular dioctylamine) were incompatible with the MS detectors. Furthermore, and in contrast to our analytical protocol, ISTD was not used in these methods.

Thus, as compared to the previously described protocols, our method has a much higher sensitivity at stable retention times, precise differentiation of the individual analytes, and better accuracy thanks to use of ISTD. On the other hand, our method requires a longer sample preparation, and is costlier because of the more expensive instrumentation as well as of the consumables. Another limitation of the current study is the lack of LR-free biological matrix to be used for calibration purposes. In our study we used a standard addition of LR approach with determination of the noise threshold. Deuterated LR standard, which is in the research pipeline in our labs, would solve this problem.

When we used this method to demonstrate its potential clinical usage, very low concentrations of LR (0.37 ± 0.17 mg/dL; 6.4 ± 2.9 μmol/L, Fig. 4) were observed in the sera of neonates treated with PT, despite a dramatic decrease in UCB concentrations. Such low LR concentrations were of the same order of magnitude as those published by Ebbesen *et al*.^27^.

To understand why LR concentrations in clinical samples are low, we performed an *in vitro* study of human serum spiked with bilirubin (the initial concentration of UCB was 18.4 mg/dL (315 μmol/L)) exposed to continuous PT. LR was produced at a 24% yield from UCB, giving LR concentrations of 4.39 mg/dL (75 μmol/L). The sum of the LR plus UCB molar concentrations accounted for 43% of the initial UCB concentration. This mass balance changed dramatically after 6 h of irradiation, when LR concentrations decreased to only 0.18 mg/dL (3 μmol/L). These data clearly indicate that LR must be further efficiently degraded to secondary photoproducts (most likely tri-, di-, and monopyrroles). Another factor accounting for low LR concentrations in the clinical samples is certainly the increased excretion of LR and bilirubin photoproducts *via* the urine and bile^28^. However, no quantitative data exist on the efficiency of LR photoproduction, its distribution among different biological compartments, or even its transfer across the blood brain barrier. Even more importantly, we do not have much data on the possible biological effects of bilirubin photoproducts, which may act in both protective as well as in harmful ways. In fact, it has recently been reported that neonates with extremely low birth weights treated with aggressive PT have higher morbidity and mortality^7–9^. In addition, as we demonstrated in our previous *in vitro/ex vivo* studies, LR, although not affecting the viability of neuronal cells^24^, can produce pro-inflammatory cytokines^19^, which may certainly account for certain of the clinical observations. These facts emphasize the need for detailed studies of bilirubin photochemistry using proper analytical tools.

In summary, we present a sensitive and accurate LC-MS/MS method for simultaneous determination of LR and UCB. This method might improve our understanding of the kinetics and biology of bilirubin photoproducts. Provided that the lack of commercial LR standard will be overcome by synthetic approach, together with increasing availability of LC-MS/MS instrumentation, this method has also clinical potential to monitor neonates on PT.

## Supplementary information

Supplementary Information.

## Data Availability

All data generated or analyzed during this study are included in this published article (and its Supplementary Information files).
