# Supplementary material for: A novel accurate LC-MS/MS method for quantitative determination of Z-lumirubin
Source: Sci Rep. 2020 Mar 10;10:4411. doi: 10.1038/s41598-020-61280-z (PMC7064611; doi:10.1038/s41598-020-61280-z)
Supplement: Supplementary file 1 — Supplementary Information. [file 41598_2020_61280_MOESM1_ESM.docx]

### **Supplementary Figures**

**Suppl. Fig. 1. Stability of UCB and LR in the human serum samples stored at -80°C for three months.**


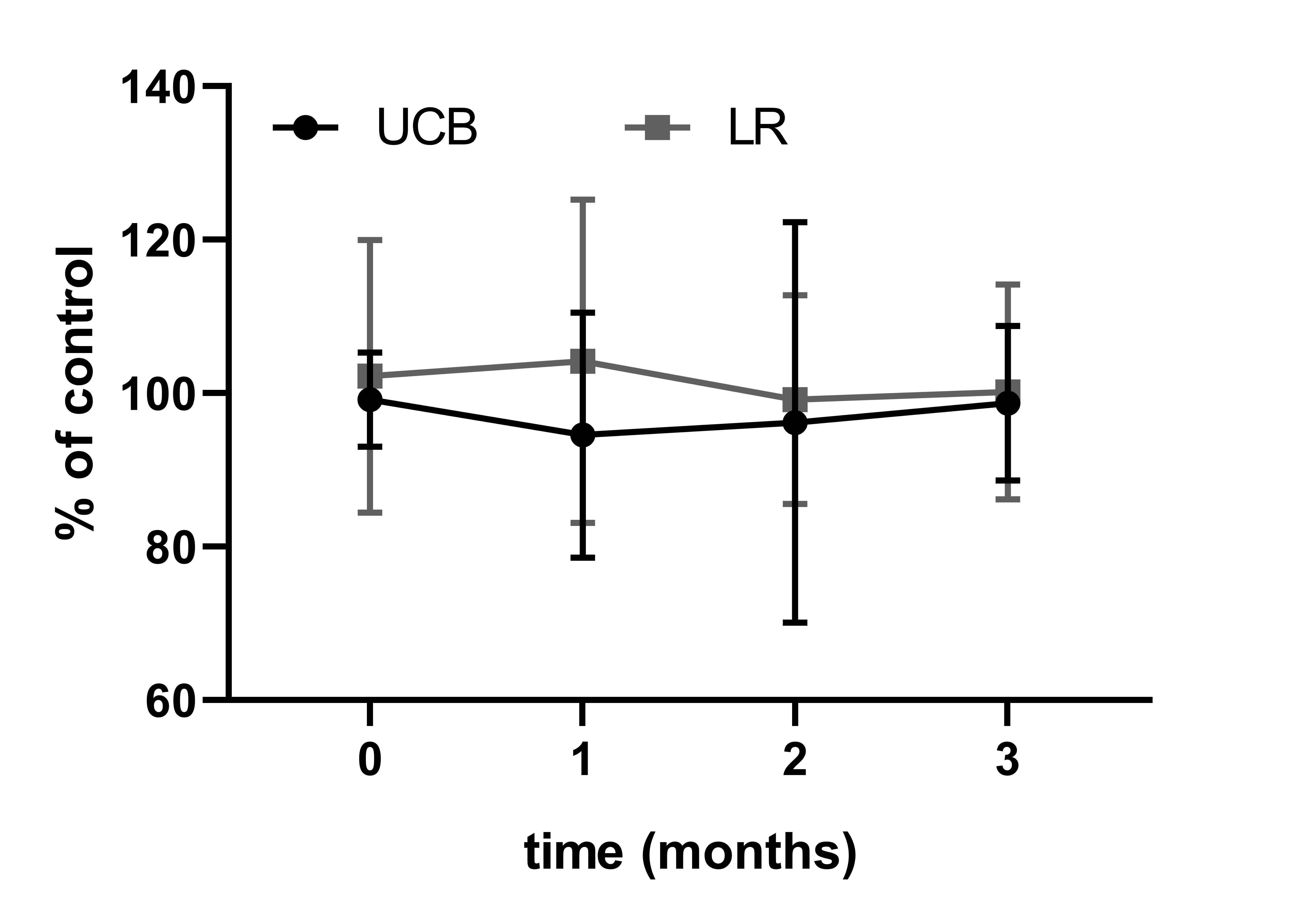


Samples of human serum (n = 6) were spiked with UCB and LR to a final concentration of 2.3 and 0.58 mg/dL (40 and 10 μmol/L), respectively, and stored at -80°C.

Relative concentrations were expressed as a mean percentage ± SD (a ratio of analyte concentration to that in the fresh sample).

LR, lumirubin; UCB, unconjugated bilirubin;

**Suppl. Fig. 2. The effect of antioxidants on LR (A) and UCB (B) determination.**

**A)**


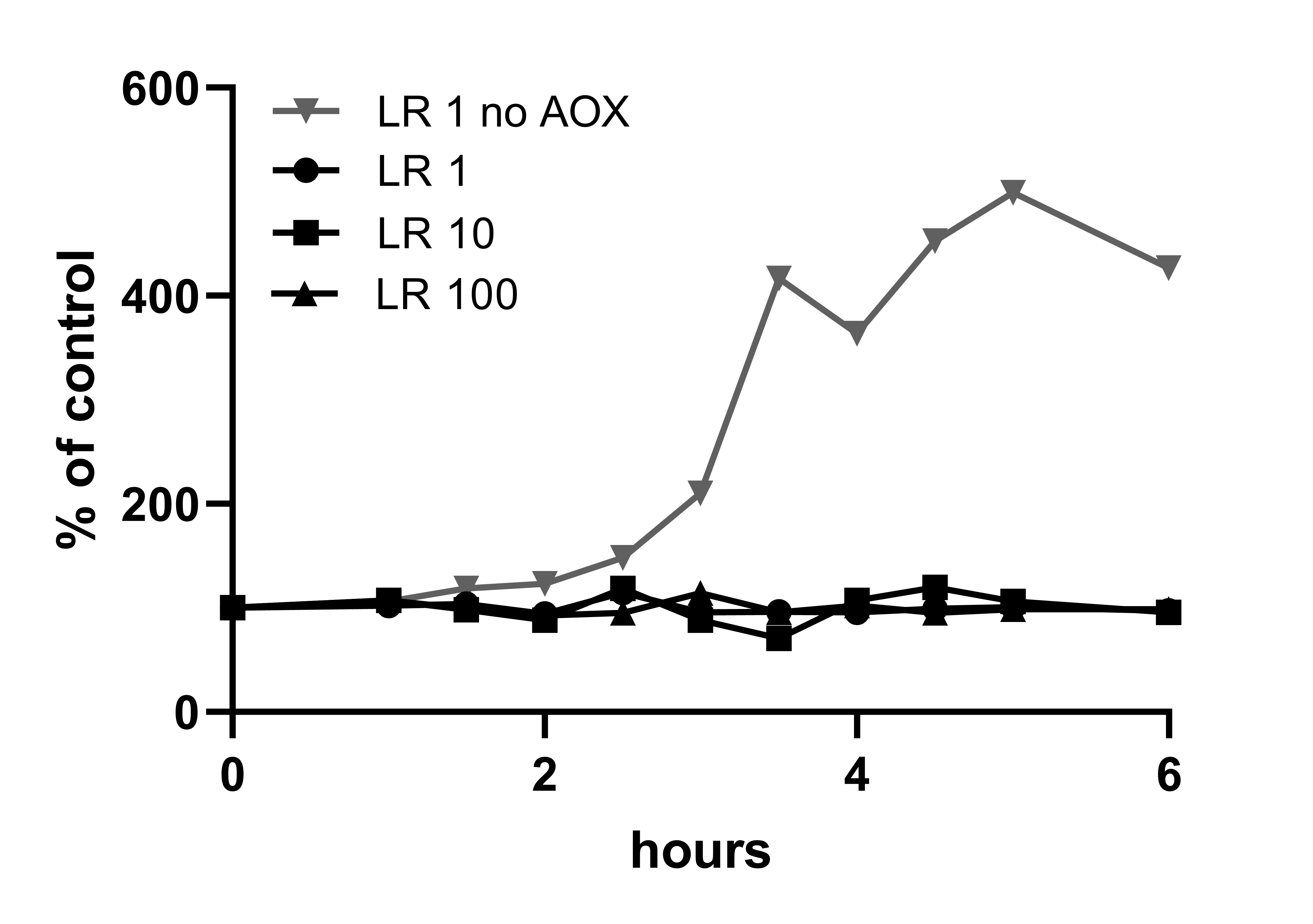


**B)**


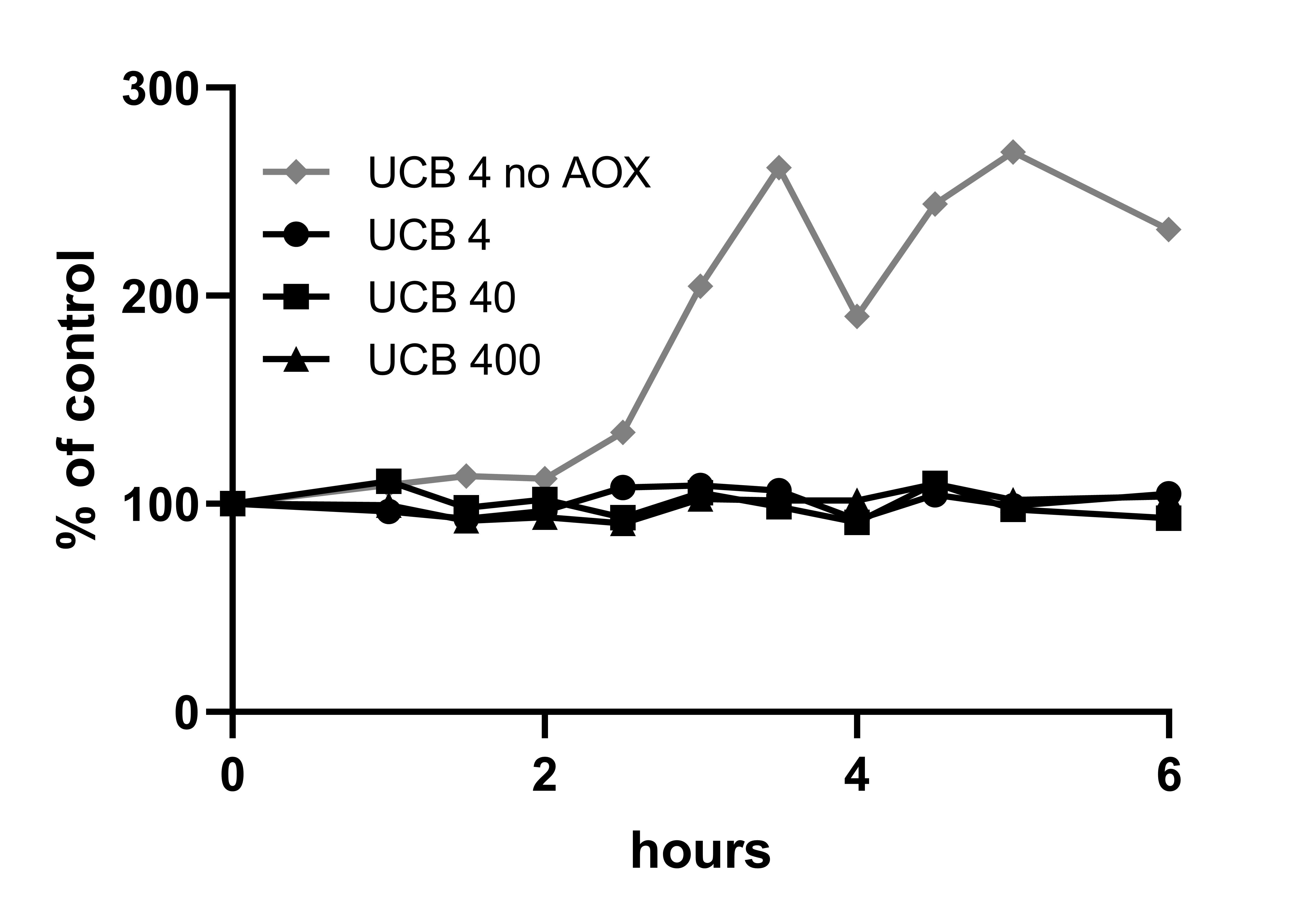


Serum samples measured during 6 h using a temperature-controlled auto-sampler (15°C). Samples were prepared and extracted by standard procedure using methanol with antioxidants (black lines), and methanol without antioxidants (grey lines).

AOX, antioxidants; LR, lumirubin; UCB unconjugated bilirubin. Numbers indicate concentrations of LR/UCB in µmol/L.
